# Editorial: Community series in neurobiological biomarkers for developing novel treatments of substance and non-substance addiction, volume II

**DOI:** 10.3389/fpsyt.2023.1134561

**Published:** 2023-04-13

**Authors:** Yehong Fang, Yi Liu, Ling Li, Dara G. Ghahremani, Jianhua Chen, Kyoji Okita, Wenbin Guo, Yanhui Liao

**Affiliations:** ^1^Department of Psychiatry, Sir Run Run Shaw Hospital, Zhejiang University School of Medicine, Hangzhou, China; ^2^Department of Psychiatry and Biobehavioral Sciences, Semel Institute for Neuroscience and Human Behavior, University of California, Los Angeles, Los Angeles, CA, United States; ^3^Shanghai Mental Health Center, Shanghai Jiao Tong University School of Medicine, Shanghai, China; ^4^Integrative Brain Imaging Center of National Center of Neurology and Psychiatry, Kodaira, Japan; ^5^National Clinical Research Center for Mental Disorders, Department of Psychiatry, The Second Xiangya Hospital of Central South University, Changsha, China

**Keywords:** neurobiological biomarkers, addiction, novel treatment, non-substance addiction, substance addiction

In 2021, we published the first special issue on “*Neurobiological Biomarkers for Developing Novel Treatments of Substance and Non-substance Addiction*” in *Frontiers in Psychiatry* that covered a wide breadth of research, such as animal studies, clinical studies, cross-section studies, and meta-analyses (1). These studies highlighted neural mechanisms, detecting biomarkers, diagnosis, and treatment responses of substance and non-substance addiction. In parallel with significant breakthroughs in neuroscience studies, recent years have seen tremendous advances in several frontiers in substance and non-substance addiction. Therefore, it is time for us to summarize the recent progress in this second special issue. A total of 13 original studies, a systematic review, and a clinical trial are included in this issue.

In the studies on nicotine addiction, Wen et al. focused on the local spontaneous brain activity of cigarette smoking and used resting-state fMRI, mainly the regional homogeneity (ReHo) to investigate the sex-related effects on the brain structural and functional changes. They found some sex-dependent differences in spontaneous brain activity, e.g., the ReHo within the left cerebellum crus1 was negatively related to craving scores in male smokers but not in female smokers. These findings further our understanding of the neuropathological (from the neuroimaging perspective) sex-specific effects of nicotine addiction. In addition, Li et al. group conducted an ecological momentary assessment study to investigate the different effects of two strategies of smoking cessation, immediate reduction and gradual reduction, on cravings. In contrast with the gradual nicotine reduction group, the immediate nicotine reduction group showed significantly lower cravings, which adds to the evidence base for reduced nicotine content in cigarettes.

Alcohol use disorder is one of the common substance use disorders in the general population (2, 3). To explore the prevalence of alcohol use, especially among elderly people, Qiu, Lv, et al. conducted a national community-based survey to derive the prevalence and correlates of risky drinking in Chinese elderly people aged 80 and over. They proposed that more attention should be given to risky drinking among very elderly people. The consensus on alcohol dependence is that both genetic and environmental determinants are involved in this chronic mental disease (4). As a result, a high prevalence of alcohol dependence is often observed in offspring who have parents with alcohol dependence. Among the offspring of parents with alcohol dependence (OPAD), Qiu, Wang, et al. investigated the personality traits and P300 component in those currently risky drinking and not risky drinking. They found P300 component was differentiated between OPAD with and without risky drinking, which indicated that P300 may be used as an early detection index of vulnerable OPAD. Indeed, given the serious dangers of alcohol dependence and smoking, numerous studies have focused on interventions and treatments for smoking cessation and alcohol withdrawal (5, 6). High-frequency repetitive transcranial magnetic stimulation (rTMS) is one of the popular non-invasive interventions and recently it has been applied in the field of addiction (7, 8). Feng et al. investigated the effect of high-frequency rTMS on the attention bias of alcohol-related cues in male patients with alcohol use disorder and obtained a positive result.

The prevalence of methamphetamine use and methamphetamine use disorder (MAUD) is a serious public health issue (9). A high level of childhood trauma and aggression is found to be associated with MAUD. Therefore, Liu et al. conducted a cross-sectional study to examine the relationship between methamphetamine-use characteristics and childhood trauma with aggression in men with MAUD. The result showed a positive correlation between high levels of childhood trauma and aggression in the MAUD population. Relapse occurs during abstinence from methamphetamine. Wang et al. team investigated electroencephalography (EEG) microstate changes in methamphetamine-dependent patients under exposure to drug-related cues in virtual reality environments.

The use of opioids is a double-edged sword. Currently, it is reported that the medicinal use of opioids in Chinese medical institutions may be quite conservative (Fang et al.). As shown in Fang et al. study, the overall level of consumption of opioids in the Second Xiangya Hospital remained relatively low, indicating the urgent necessity for increasing the availability of opioids. However, overprescribing opioids can lead to the development of iatrogenic addiction and finally become an opioid use disorder. Opioid use disorder is a chronic relapsing disorder. Liu et al. and Wang et al. group investigated the peripheral mechanism of opioid use disorder by conducting a whole transcriptome sequencing of peripheral blood and found that GnRH/PI3K/Akt signaling is associated with opioid use disorder.

As for betel quid dependence (BQD), Chen et al. research team also focused on the brain's spontaneous neural activity. In this study, they investigated changes in BQD chewers using a new method called the percent amplitude of fluctuation (PerAF). Brain regions such as the right anterior cingulate cortex (ACC), right middle frontal gyrus (MFG), right insula, right precuneus, left putamen, left supramarginal gyrus (SMG), and left cerebellum were found to have decreased PerAF, and the right orbitofrontal and left superior temporal gyrus (STG) were found to have increased PerAF in BQD chewers. It will be of great interest to test PerAF as a potential sensitive biomarker for identifying spontaneous brain activity changes in other addiction disorders.

Amphetamine shows severe abuse potential and amphetamine withdrawal is a thorny issue. To investigate the cognition improvement differences between d-amphetamine and its prodrug lisdexamfetamine, Chen et al. compared the effects of d-amphetamine (i.p) and lisdexamfetamine (p.o) at equimolar doses by observing rat's behaviors on locomotion, spatial working memory, and recognition memory. The results showed that, in the medial prefrontal cortex (mPFC), lisdexamfetamine leads to a steady and lasting dopamine release pattern and thus shows more effectiveness than d-amphetamine in improving spatial cognitive performance.

Non-substance addiction, especially gaming disorder (GD) has been recognized as an official diagnostic entity in the latest revision of the International Classification of Diseases (ICD-11) (10), but a suitable localized diagnostic tool in Chinese is still missing. Zhang et al. team developed a new tool (the ICD-11 Gaming Disorder Symptom Questionnaire) and examined the effectiveness of identifying individuals at risk of GD both in medical and non-medical settings. Effective screening and detection as well as early intervention are vital for preventing GD. Li et al. investigated escapism-based motivation (EBM) using the eye-tracking analysis in high-risk internet GD (HIGD) students and found EBM may be a potential negative indicator in the HIGD participants as for its significant association with impulsivity, self-emotion management ability, and response inhibition. As for the treatment of non-substance addition, Dai et al. investigated the use of electroacupuncture (EA) and psychotherapy (PT) and observed the effective results in patients with pathological internet use. Furthermore, they found that monoamine oxidase type A (MAOA) may serve as the underlying mechanism of psychotherapy for internet addiction.

The relationship between obesity and gray matter volume (GMV) alterations is still a point of concern. By conducting a meta-analysis of voxel-based GMV, Wang et al. group compared the GMV changes in overweight and obese subjects and lean controls. Compared with lean controls, lower GMV in the left putamen and right precentral gyrus was observed in individuals with overweight and obesity. These results suggested that the structural basis for reward processing and sensorimotor processing may be dysregulated in overweight and obese subjects.

In summary, the second special issue presented here will further our understanding of substance and non-substance addiction. Our ultimate goal was to understand addiction better and to develop more efficacious and safe treatments for addiction.

## Author contributions

YF and YLiu contributed to the conception and design of the editorial. YLiao and LL contributed considerably to the writing. DG, JC, KO, and WG contributed to the revision of the editorial. All authors contributed to the article and approved the submitted version.
